# The advantage of ultrasonography in the diagnosis of extracranial vertebral artery dissection: Two case reports

**DOI:** 10.1097/MD.0000000000006379

**Published:** 2017-03-24

**Authors:** Lijuan Yang, Haitao Ran

**Affiliations:** a Institute of Ultrasound Imaging, Chongqing Medical University, Chongqing, China; b Department of Neurology, Baotou Central Hospital, Baotou, Inner Mongolia, China; c Department of Ultrasonography, The Second Affiliated Hospital, Chongqing Medical University, Chongqing, China.

**Keywords:** cervical artery, ultrasonography, vertebral artery dissection

## Abstract

**Rationale:**

With the rapid development in technology of vascular imaging, the detection of artery dissection is also gradually increasing. Cervical artery dissection, including carotid artery and vertebral artery dissection, is associated in at least 20% of young adult patients with stroke. So, the diagnosis of cervical artery has become a great challenge.

**Patient concerns:**

We reported 2 patients who complained of dizziness and pain, the findings of US confirmed the presence of extracranial vertebral artery dissection.

**Diagnoses:**

The floating membrane in lumen and intramural hematoma were found in US, consistent with vertebral artery dissection, whereas DSA revealed no typical sign of artery dissection.

**Interventions:**

In order to the definite diagnosis we persuaded the patients to undergo DSA, but there was no strong evidence on the diagnosis of vertebtal artery dissection.

**Outcomes:**

The patients were diagnosed of vertebral artery dissection by US.

**Lessons:**

US show more advantages in diagnosis of extracranial vertebral artery dissection, might even be considered as the first choice.

## 1 Introduction

In view of the diverse clinical manifestations, the diagnosis of vertebral artery dissection greatly depends on imaging examination. But the imaging diagnostic criteria have still not reached a consensus due to different available methods. In this study, we used the standard ultrasonography (US) for diagnosing ex-vertebral artery dissection,^[1]^ which included multiple segmental intramural hematoma, the membranous tube cavity of the echo, double cavity structure, and irregular artery stenosis. Of these, only 1 or any 1 can be a definitive diagnosis. With high-resolution ultrasound instrument, the diagnostic accuracy of artery dissection is unprecedentedly increasing, but most clinicians relied on digital subtraction angiography (DSA) and US has been often overlooked. This study allows clinicians to understand more about US and know its importance clinically, especially in diagnosing extracranial vertebral artery dissection.

## 2 Case report

The case report was approved by the Ethics Committees of Baotou Central Hospital, China, and all participants provided written informed consent.

### 2.1 Case report 1

A 72-year-old woman was presented to the neurology department with dizziness that lasted for almost 3.5 h. Apart from the presence of bilateral Babinski signs, the patient had no other neurological symptoms or signs. The patient had a history of high blood pressure and diabetes and no other significant pathology was observed. The patient was under treatment for diabetes. Physical examination was unremarkable. Arterial blood pressure was 150/90 mm Hg, pulse was 88 rpm, respiratory rate was 21 bpm, and temperature was 36.8°C. Magnetic resonance imaging (MRI) of brain, carotid ultrasound, and DSA were performed. MRI revealed acute infarction in the left occipital areas and bilateral basal ganglia, frontal, parietal multiple lacunar infarction, and some softening of the brain tissues. DSA showed left vertebral artery occlusion. Carotid ultrasound showed left vertebral artery occlusion caused by dissection (the floating membrane in lumen of initial segment).

### 2.2 Case report 2

**Figure 1:**
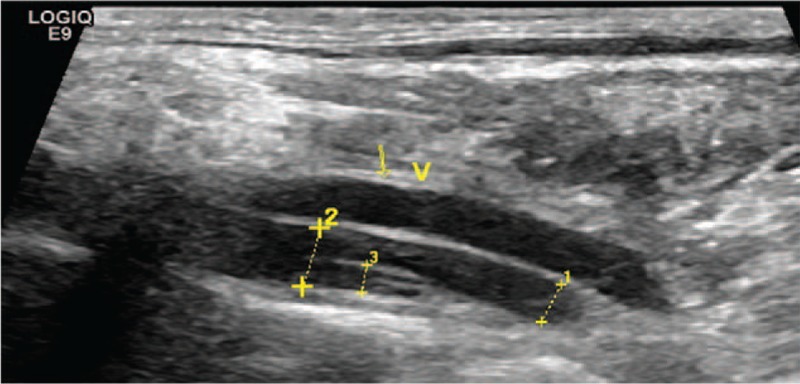
B-mode shows the intimal flap in the lumen of the left vertebral artery.

**Figure 2:**
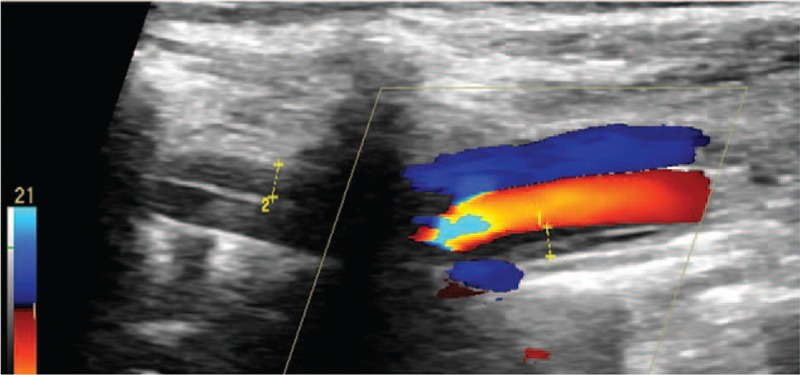
Color Doppler imaging shows intramural hematoma in the vertebral artery of initial segment.

**Figure 3:**
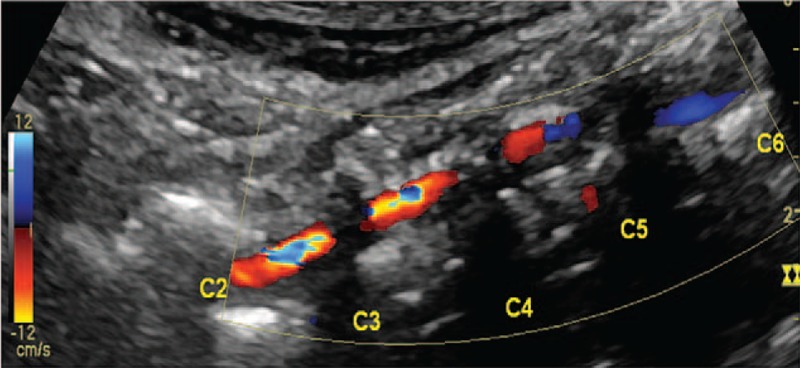
Color Doppler imaging shows intramural hematoma in the vertebral artery of the intervertebral segment.

**Figure 4:**
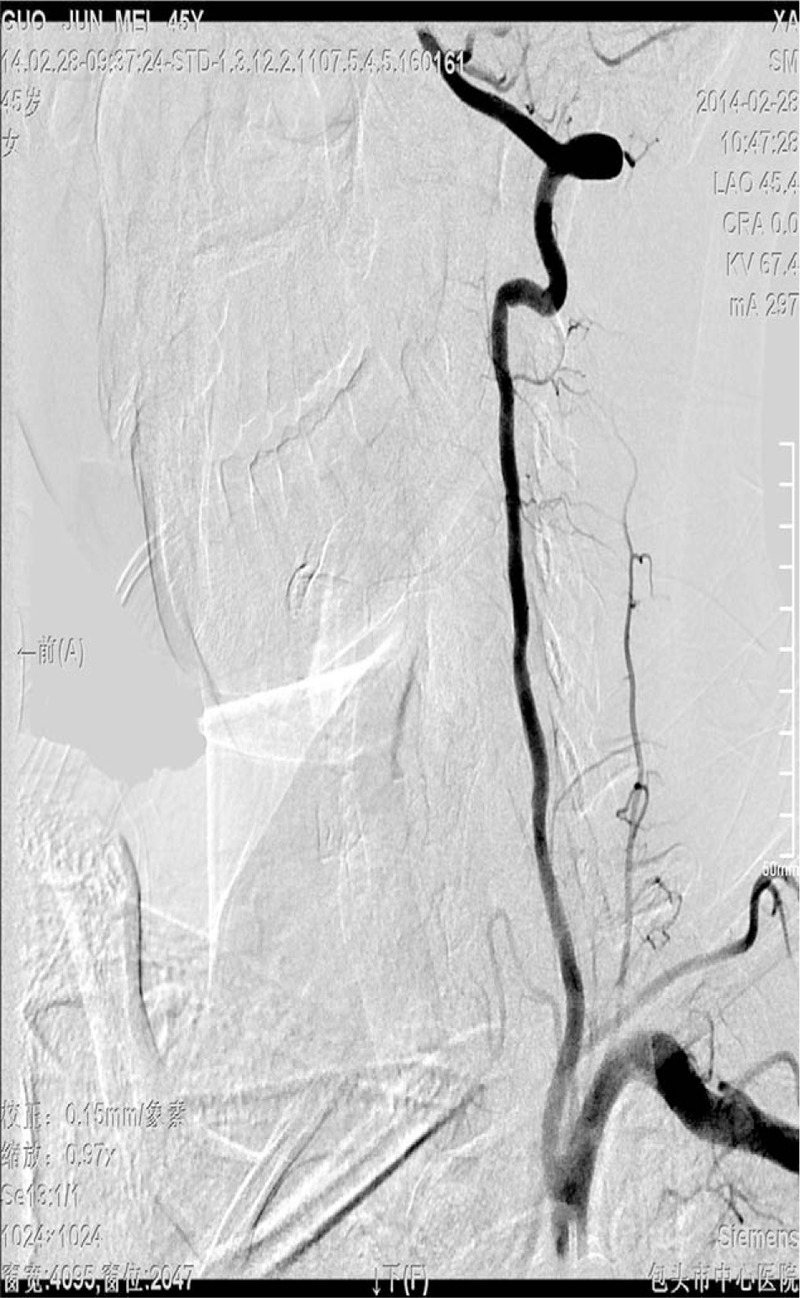
Digital subtraction angiography shows the left vertebral artery.

A 45-year-old woman was presented to the orthopedic department with dizziness and pain complaints with trapped cervical acid which started 20 days ago. She underwent cervical spine X-ray, which showed hyperplasia of the cervical bone. She then underwent carotid US which showed left vertebral artery dissection (intramural hematoma) (Figs. 1–3). In order to further examine, the patient was advised to the neurological department, where DSA was performed which showed normal left vertebral artery without any evidence of dissection (Fig. 4).

## 3 Discussion

### 3.1 Pathophysiology of artery dissection

Artery dissection generally arises from a tear in the vessel wall which then allows the blood under arterial pressure to enter the wall of the artery, either into subintimal or into subadventitial. Signs appear most commonly in the former place, which results in artery stenosis or occlusion, and occurrence of signs in the later area is rare, which often results in aneurysmal dilatation. Tearing of membrane divides the artery into false lumen and true lumen. If there are 2 tears in the dissection artery, the blood from the true lumen can enter into the false lumen from 1 tear and then reflow into the true lumen from the other tear, namely the flow direction in the false lumen is the same as true lumen. If there is only 1 tear, the flow in the false lumen may move in a to and fro manner. Both of the above pathotypes were present in the double lumen in the artery dissection on US images. However, artery dissection most often presents as intramural hematoma on US images, namely the false lumen is full of hypoecho and lack of signal flow, in other words, the artery dissection has a thickened and mainly hypoechogenic wall. Some people assume that the to and fro signal flow in the false lumen can easily form into thrombus, and hence it is known as hematoma. This is composed of thrombus, and therefore intramural hematoma cannot be considered very accurate. One study reported that the thickened wall was composed of hematoma and intraluminal thrombus.^[2]^ Another study reported that the rupture of vasa vasorum was filled with bleeding within the media which resulted in the separation of vessel wall layers and formation of hematoma in the false lumen, intima may then be disrupted by the rupture of primary intramural hematoma into the arterial lumen.^[3,4]^ So, some dissections of cervical arteries may be caused by primary intramural hematoma. But the current medical research methods are unable to demonstrate the mechanism of hematoma formation and distinguish primary tear from primary intramural hematoma. Additionally, artery dissection can also present the aneurysmal dilatation, which is different from aneurysm and pseudoaneurysm. A tear in the vessel wall resulted in the abnormal structure formation of the vessel wall which precipitates aneurysmal dilatation and its further rupture often resulted in the intracranial subarachnoid hemorrhage. Thus, aneurysm dissection should be more accurate.

### 3.2 Imaging diagnosis of artery dissection

Artery dissection is not uncommon in young adult patients with stroke. Although the etiology and pathophysiology are still not very clear, various clinical manifestations and number of standardized treatments were present. The neuroimaging diagnosis should be performed by MRI, computerized tomographic angiography (CTA), US, and DSA. Each method has its own advantages and disadvantages. The accuracy of each method varies according to the location and pathotype of the dissection. DSA has long been regarded as the gold standard for the diagnosis of dissection, but it lacks in visualizing the arterial wall or mural hematoma, so the diagnosis cannot be always accurate. Additionally, when the artery is occluded, DSA revealed no information about the lumen of the artery in addition to the diagnosis of occlusion. However, US can make full use of its advantages in analyzing the reasons of occlusion in accordance to the wall and lumen. With the continuous improvement of equipment and diagnosis level, reliability of US diagnosis in artery dissection is gradually accepted by people, US has been recommended in the initial diagnosis and follow-up evaluation of carotid artery and vertebral artery dissections. Magnetic resonance angiogram (MRA) combined with T1-weighted axial cervical MRI scans with fat-saturation technique due to its high sensitivity and specificity and the absence of irradiation has been recommended for the diagnosis of carotid artery dissection and vertebral artery dissection.^[5]^ However, when the dissection was located in the vertebra, the accuracy of diagnosis was not superior to CTA. In addition, the contraindications and high cost limit the wide application of MRI and MRA. CTA is usually done only when there is a contraindication or difficulty in access to MRI because of its high radiation exposure.

## 4 Conclusion

We reported 2 cases of extracranial vertebral artery dissection onset and the process of diagnosis. In case 1, due to the neat intramural hematoma and appropriate true lumen, no significant findings were found on DSA. Whereas, the performance of US in diagnosing intramural hematoma was obvious, and the diagnosis was clear. In case 2, in view of the patient's age, disease history, and stroke caused by atherosclerotic vascular occlusion benefitted to easily diagnose which was also confirmed by DSA that was presented in the left vertebral artery occlusion. But the slightly floating membrane in the lumen of the left vertebral artery on US suggested that the left vertebral artery occlusion resulted from a primary dissection.

Artery dissection, due to lack of typical clinical manifestations, is often misdiagnosed or missed diagnosis. The confirmation of vertebral artery dissection mainly relies on imaging studies. DSA because of its invasive nature is replaced by noninvasive examination methods gradually. Of which, US has been chosen because of its noninvasive, safe, low cost, and almost no contraindications, which showed more advantages compared with other diagnostic imaging methods and patients with vertebral artery dissection can benefit from US more than what they expected.
